# A Nanothermodynamic Approach to the Shuttleworth and Lippman Equations

**DOI:** 10.3390/e28060645

**Published:** 2026-06-08

**Authors:** Claire Chassagne, Dick Bedeaux, Signe Kjelstrup

**Affiliations:** 1 Section of Environmental Fluid Mechanics, Department of Hydraulic Engineering, Delft University of Technology, 2628 CN Delft, The Netherlands; 2 PoreLab, Department of Chemistry, Norwegian University of Science and Technology, NTNU, NO-7491 Trondheim, Norwaysigne.kjelstrup@ntnu.no (S.K.)

**Keywords:** nanothermodynamics, integral and differential surface tension, surface excess variables, Helfrich curvature energy, subdivision potential, Shuttleworth equation, Lippman equation

## Abstract

The Shuttleworth and Lippman equations are well-known equations used to link surface tension and stress (Shuttleworth) and surface tension and electric surface potentials (Lippmann). We show that the Shuttleworth and Lippman equations have a common thermodynamic basis, common to systems that possess a relatively large interfacial energy. This is relevant for problems of droplet stability, colloidal suspensions, electrode surfaces and more. Both equations are derived for systems that are not Euler homogeneous in the manner classical systems are. Hill’s thermodynamics for *small* systems is used to address this problem. *Small* in this context refers to systems with interfacial energies that are size- or shape-dependent. The resulting Hill–Gibbs–Duhem equation, an extension of the classical Gibbs–Duhem’s equation, gives the common basis for the Shuttleworth and Lippman equations. Hill’s thermodynamics enables us to rigorously define two types of surface tension, the *differential surface tension* and the *integral surface tension*. These surface tensions are linked by the system’s *subdivision potential*. From Helfrich’s equation we obtain a scaling law for the subdivision potential as function of the interfacial curvature. The dependence of the resulting subdivision potential on the system curvature is predicted. A critical analysis of the literature about the Shuttleworth and Lippman equations is given.

## 1. Introduction

The surface tension is a parameter that appears in the Shuttleworth [1] and Lippman equations [2]. The Shuttleworth equation relates the surface tension to the surface stress, while the Lippman equation relates the surface tension to the surface electric potential (or surface charge density). The equations have been used to relate surface properties of neutral and charged systems, principally at solid/fluid surfaces. As debates in the literature have shown [3,4,5,6,7], care should be taken to properly define these properties and their domain of validity.

The term “surface tension” is mentioned twice by IUPAC. Under the headline “surface tension”, it is defined as the “work required to increase a surface area divided by that area” (the first definition) [8]. Under the headline “surface work”, the surface tension is defined as “the intensive factor in the differential expression for the work required to increase the area of the surface. Measured under reversible conditions at constant temperature (and normally constant pressure) and referred to unit area, this work the so-called differential work is equal to the static surface tension” (the second definition) [9]. Only in the second, but not the first definition, is reference made to work performed under reversible conditions. The minimum work performed at constant temperature, *T*, and pressure, *p*, is identified by a change in Gibbs energy. Butt et al. and Fletcher et al. give a good overview of the concepts and their definition [4,10]. These authors are dealing with systems in the thermodynamic limit.

Fletcher et al. [4] define the surface tension γ at constant T,p as the derivative of the excess Gibbs energy, $G^{s}$, of the surface Ω:

(1) $$\gamma ={\left(\frac{\partial G^{s}}{\partial \Omega }\right)}_{T,p}$$

The definition given by Equation (1) differs from the first IUPAC definition, but it agrees with the second IUPAC definition when work is done along a reversible path. Knowing the variation in $G^{s}$ with Ω, we find the surface tension from Equation (1) as the derivative of $G^{s}$ with respect to Ω at constant T,p. Fletcher et al. remarks next that an isotropic solid has an area-dependent excess Gibbs energy. They define the surface excess Gibbs energy density by $\hat{G}=G^{s}/\Omega$. It follows that

(2) $$\gamma ={\left(\frac{\partial G^{s}}{\partial \Omega }\right)}_{T,p}={\left(\frac{\partial (\hat{G}\Omega )}{\partial \Omega }\right)}_{T,p}={\left[\hat{G}\left(\Omega \right)+\Omega \frac{\partial \hat{G}\left(\Omega \right)}{\partial \Omega }\right]}_{T,p}$$

The second IUPAC definition refers to $\hat{G}$ with the assumption that it is an intensive variable, implying that $\hat{G}$ cannot depend on Ω. Both IUPAC definitions therefore make the assumption that $\partial \hat{G}\left(\Omega \right)/\partial \Omega =0$ and that, consequently, $\gamma =\hat{G}$. The IUPAC definition of the surface tension is in this sense only a particular case of Equation (2). It is correct for some types of surfaces, but it is not correct, for example, for elastic solids for which $\partial \hat{G}\left(\Omega \right)/\partial \Omega \neq 0$. Equation (2) has the same form as the equation proposed by Shuttleworth in order to explain experiments on stress in crystalline and elastic surfaces [1] and bears his name. The Lippman equation [10,11] is similarly used without considering that the excess surface energy $G^{s}$ depends on system size.

Equations like the Shuttleworth equation, as well as the Lippman equation, are traditionally derived for systems that are in the thermodynamic limit. Such systems are characterized by functions that are Euler homogeneous [12] of the first order. A function *M* of the variable set *X* is Euler homogeneous of the order *n* in *X*, when *M* obeys [12]

(3) $$M\left(\lambda X\right)=\lambda^{n}M\left(X\right),$$

where λ is an arbitrary constant. For example, the internal energy *U* of a system is a Euler homogeneous function of degree n=1 in the variables *V* (volume) and *N* (number of particles). The value of *U* will double when the system volume or number of particles doubles. The temperature *T* and pressure *p* are functions of degree zero (n=0). Their values remain the same when the system size doubles.

The expression for the surface tension has led to confusion in the past. The reason is that small system properties also depend on the *surface curvatures* of the system, a fact often overlooked. The surface tension will then depend on the curvatures of the surface between two bulk phases. When the area of this surface, Ω, is changed, while the volume of one of the phases is kept constant, the curvatures of the surface and, therefore, the value of the surface tension may change. The surface energy is then a function of the surface curvatures. It possesses energy forms that are not proportional to the volume or to the surface area. Consequently, the system has variables that are no longer Euler homogeneous of degree one (n=1).

The question now is how the classical equations change when the functions U,G, etc., are no longer Euler homogeneous in the variable Ω. An answer to this question is important, because systems that possesses surface energies are central in energy conversion [13].

Hill’s thermodynamics for small systems [14] offers a systematic approach that can help. Hill called his approach *small system thermodynamics* or *nanothermodynamics* [14]. *Small* in this context is a system with significant interfacial (surface) energy, of the order, say, of the energy of one of the bulk phases surrounding it. Such a system could be a *small* colloidal sphere, an example we will use in the next section. Interfacial energy is contained in surface curvatures. Hill’s original theory was recently extended by Bedeaux and coworkers [15,16,17,18,19,20] to describe energy changes in small systems. Using Hill’s theory, it is possible to derive Equation (2) in a more systematic manner and extend the range of validity of the Shuttleworth and Lippman equations. In particular, we shall see how surface energies connected to surface curvatures can be described. The analysis will in this manner expand the state of the art [4] and clarify the definitions above.

The approach presented in the article is perfectly general and can be used for all kinds of droplets or bubbles surrounded by their own vapor of another fluid. We have chosen as an example a suspension of hard colloidal spheres immersed in a fluid, which is justified under the assumptions that the surface free energy and surface stress are isotropic and that the spheres are in a homogeneous state of hydrostatic stress [21].

The paper has the following outline. We present the theory of Hill extended to an isotropic spherical phase in a fluid in Section 2, using the surface area, Ω, as a variable. The other control variables are the temperature (*T*), the solution pressure (*p*) and the chemical potentials ($\mu_{k}$). We derive a Shuttleworth(-like) equation using the principles of small system thermodynamics. In this approach, we define and then use an integral and a differential surface tension, $\hat{\gamma }$ and γ, respectively. Their difference gives the subdivision potential, ε, defined by $\gamma -\hat{\gamma }=\varepsilon /\Omega$. In Section 3, we consider a charged colloidal particle in an electrolyte solution and show how Hill’s theory enables us to derive the Lippman equation for this system. A discussion of the literature regarding the Shuttleworth and Lippman equations is given in Section 4.

## 2. Theory

The generic system that we propose to study possesses energy forms that are neither proportional to the system volume nor to the surface area. This system is composed of two bulk phases separated by an interfacial region, and it is the interfacial region, through the energy of curvature, that leads to variables that are not Euler homogeneous of degree one. The *thermodynamic theory of small systems* by Hill [14] is particularly well suited for the analysis. This theory, extended by Bedeaux and coworkers [15,16,17,18,19,20], offers an opportunity to re-formulate the Gibbs equation for variables that are no longer Euler homogeneous of degree one. The analysis and the results are relevant for all systems that are small, in the sense that their interfacial energy is significant compared to the energy of one of the bulk phases.

**Figure 1:**
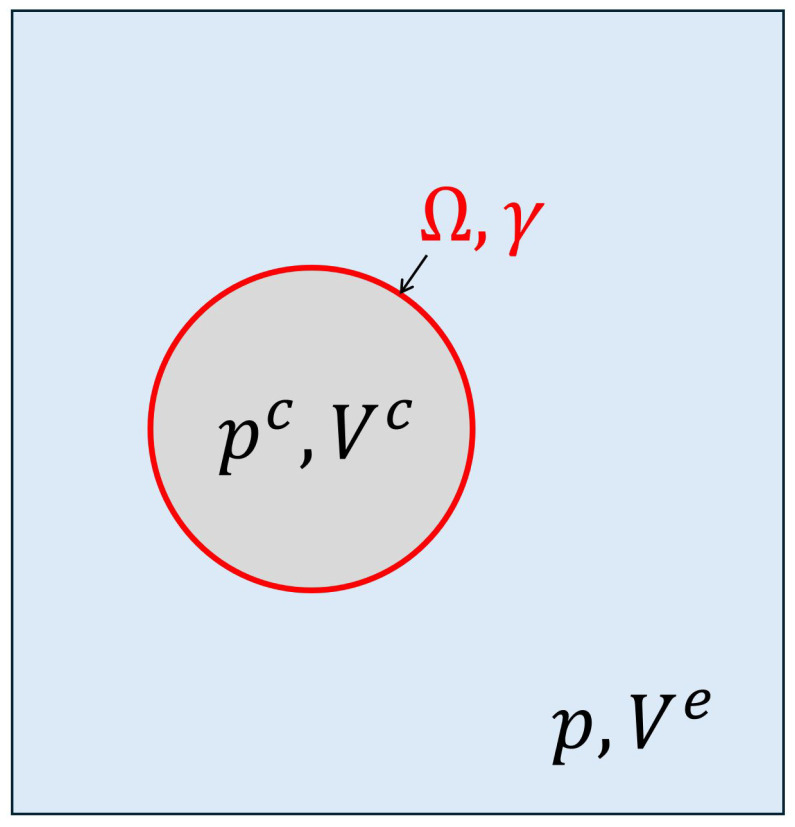
A colloidal particle of volume $V^{c}$ in an electrolyte solution of volume $V^{e}$ separated by a surface Ω associated with a surface tension γ. The volume $V=V^{c}+V^{e}$ in blue and gray is the volume of what is defined as a single replica. The pressures $p^{c}$ and *p* are pressures of the colloidal and electrolyte phases, respectively. The hydrostatic pressure of the bulk fluid is controlled by a pressure reservoir, and hence, $p^{e}=p$.

Here, we consider a spherical colloidal particle of radius *a*, suspended in an electrolyte with a total volume *V*. The system is illustrated in Figure 1. The particle can be fluid or solid. In the latter case, the assumptions made are that the surface free energy and surface stress are isotropic and that the spheres are in a homogeneous state of hydrostatic stress [21]. There is a surface of area Ω between the bulk phases in question. The phases are denoted *c* (the colloidal particle) and *e* (the electrolyte). The volumes $V^{c}$ and $V^{e}$ make up the total volume $V=V^{c}+V^{e}$. The hydrostatic (external) pressure, *p*, determines the pressure of the electrolyte solution. The surface has a surface tension γ. The Young–Laplace equation applies for this system under quite general conditions [22]:

(4) $$p^{c}=p+2\gamma /a$$

Here, $p^{c}$ and *p* are pressures of the bulk phases *c* and *e*, respectively. The equation was found to be equivalent to an expression given by Hill’s thermodynamic theory, when applied to a bubble in a slit pore. The equilibrium at the interface was characterized by a constant value of the integral pressure or the effective pressure that adds surface contributions to the bulk pressure [13,19,23]. Note that Equation (4) is usually obtained by a force balance and minimization of surface energy and that the dependence of the surface tension on curvature is not explicitly discussed [24]. A length scale, for which the Hill theory will be relevant, is given by the particle radius, *a*. When *a* is large compared to 2γ/p, we have in good approximation $p^{c}=p$. The surface curvature 2/a is then negligible, and the system is large (i.e., in the thermodynamic limit). When *a* is of the same magnitude as 2γ/p, the surface curvature leads to variables that are not Euler homogeneous of degree one.

Systems that are relevant for the present study belong to the grand canonical ensemble. A subset of system variables contains the *control* variables. In the grand canonical ensemble, they are the temperature *T*, volume *V*, chemical potentials $\mu_{k}$ and surface area Ω. Alternatively, rather than controlling *V*, one may control the pressure *p*. In a following subsection, we will show how the equations are modified if *p* is controlled rather than *V*. The *k* stands for the independent components (material of which the particle is made, electrolyte) needed to make up the system. To start, we consider uncharged colloidal particles. The case of charged colloidal particles will be discussed in a forthcoming section. To deal with variables that are not Euler homogeneous, the approach of Hill and others [14,17] is to construct an ensemble of N replicas of the single system and introduce Euler homogeneity in the number N of replicas of the ensemble [14]. An example of a replica is shown in Figure 1. This replica is composed of three important elements: the colloidal bulk phase, defined by its volume $V^{c}$ and pressure $p^{c}$, the electrolyte bulk phase, defined by its volume $V^{e}$ and pressure $p^{e}$, and the interfacial region between the two bulk phases, defined by a surface area Ω and a surface tension γ. The colloidal particle is contained in the volume such that the electrolyte at the boundaries of the replica has bulk properties (i.e., the colloidal particle is far from the boundaries of the replica). The derivations presented in this section are general, and the sphere can be exchanged by a particle with any size and curvature [15,16] embedded in a fluid.

### 2.1. Thermodynamics of an Ensemble of Replica’s

The analysis starts by writing the total differential of the internal energy, $U_{t}$, for the ensemble of replica’s [17]:

(5) $$dU_{t}=TdS_{t}-pdV_{t}+\mu_{k}dN_{k,t}+\gamma d\Omega_{t}+\varepsilon dN$$

where $S_{t}$ is the total entropy and $V_{t}$ the total volume of the ensemble. The total number of unpolarized molecules or atoms of component *k* is $N_{k,t}$. We use the sum convention over double subscripts. Subscript *t* (for “total”) refers to an ensemble value for N replicas. We choose for now that $V_{t}$ and $\Omega_{t}$ are independent variables and discuss below how we replace the volume $V_{t}$ by *p* as variable, while keeping the surface area as a variable. The first four terms on the right-hand-side of Equation (5) are terms in the classical description of Clausius and Gibbs. The last term in Equation (5) is due to Hill. The equation was therefore named the Hill–Gibbs equation by Bedeaux et al. [17].

The system properties are additive in the sense that the entropy is the sum of the entropies of the colloidal and electrolyte phases plus the surface excess entropy and so on. The subdivision potential ε was introduced by Hill and is defined by

(6) $$\varepsilon \equiv {\left(\frac{\partial U_{t}}{\partial N}\right)}_{S_{t},V_{t},N_{k,t}\Omega_{t}}$$

The subdivision potential ε deals with energy contributions to $U_{t}$ that depend on the curvature and size. It corresponds to the internal energy added to the ensemble by adding one more replica, keeping the total entropy, volume, amount of components, and surface area constant, as the definition states.

The introduction of an ensemble of systems is not peculiar in thermodynamics. In fact, it is common in classical thermodynamics to use ensembles in the construction of variables. For a large system, the ensemble of replicas becomes the usual ensemble in statistical mechanics. For large systems, the subdivision potential ε is zero. Small systems, on the other hand, have ε≠0. Special for the system is that the volume, as well as the surface area, can be controlled independently of one another.

However, the ensemble variables of $U_{t}$ are not practical for descriptions of experiments; so, we want to replace $U_{t}$ with the Gibbs energy $G_{t}$. This is done by a Legendre transform on the ensemble level, $G_{t}\equiv U_{t}-TS_{t}+pV_{t}$. The total differential in the Hill–Gibbs equation for $dG_{t}$ is

(7) $$dG_{t}=-S_{t}dT+V_{t}dp+\mu_{k}dN_{k,t}+\gamma d\Omega_{t}+\varepsilon dN$$

This replaces $S_{t}$ and $V_{t}$ with T,p as variables. The Gibbs energy of the ensemble, $G_{t}$, contains the compressional energy of all ensemble members ($\gamma \Omega_{t}$).

We integrate using the Euler homogeneity of degree one in N, the number of replicas, and degree zero in the temperature and pressure. This gives

(8) $$G_{t}=\mu_{k}N_{k,t}+\gamma \Omega_{t}+\varepsilon N$$

The single system average properties ($G,S,V,N_{k}$ and Ω) are of interest: $G=G_{t}/N$, $S=S_{t}/N$, $V=V_{t}/N$, $N_{k}=N_{k,t}/N$, $\Omega =\Omega_{t}/N$. Introducing these single system values in Equation (7) and differentiating the product NG, we obtain

(9) $$NdG+GdN=-SNdT+VNdp+\mu_{k}NdN_{k,t}+\mu_{k}N_{k}dN+\gamma Nd\Omega +\gamma \Omega dN+\varepsilon dN$$

The area can also be varied by varying N, as expressed by the next-to-last term in this equation. By rearranging terms, we obtain

(10) $$\begin{array}{c} G=\mu_{k}N_{k}+\gamma \Omega +\varepsilon \end{array}$$

and

(11) $$\begin{array}{c} dG=-SdT+Vdp+\mu_{k}dN_{k}+\gamma d\Omega \end{array}$$

Equation (11) applies to one replica (the single system, as illustrated in Figure 1). Here, we make the important remark that, while Equation (11) has the appearance of the classical Gibbs equation, it relates thermodynamic quantities that are not Euler homogeneous in their size dependence.

From Equation (11), we obtain the expression for the *differential surface tension*:

(12) $$\gamma \left(T,p,N_{k},\Omega \right)={\left(\frac{\partial G}{\partial \Omega }\right)}_{T,p,N_{k}}$$

The variables to keep constant in the operation are T,p and $N_{k}$. The colloid pressure $p^{c}$ is a dependent variable, computable from p,γ and *a* by Equation (4).

**Figure 2:**
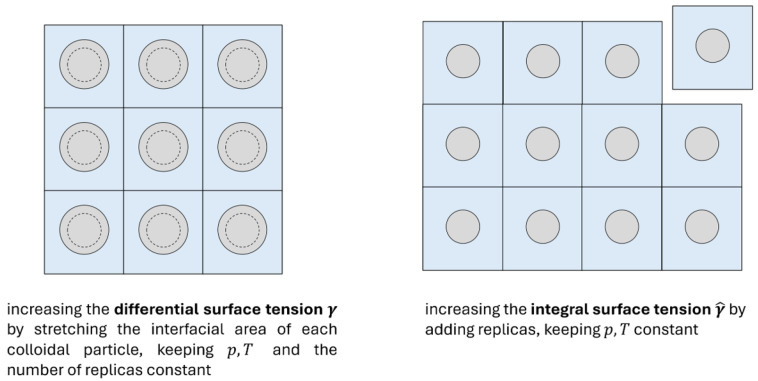
The two ways to vary the total surface area of the spheres in the replicas. One is by surface stretching (**left**) and the other by adding replicas to the system (**right**). The integral surface tension $\hat{\gamma }$ and differential surface tension γ are related to the way of varying the surface area. They are linked through the subdivision potential ε by Equation (15).

We can vary the total area $\Omega_{t}$ in two ways; see Figure 2. The area can be varied by stretching the available area, cf., the left-hand side of the figure and Equation (12). This leads to the definition of the differential surface tension, γ. The area can also be increased by adding a replica as illustrated on the right-hand side of the figure. We will see how this leads to the definition of the integral surface tension, Equation (17).

The definition of the differential surface tension given in Equation (12) is different from the one adopted by other authors and given in Equation (1). In particular, the Gibbs energy referred to in Equation (12) is the *replica’s ensemble average Gibbs energy* and not the excess Gibbs energy of the surface, as was assumed in Equation (1).

We are seeking more detailed expressions for the surface and consider the single contributions from the parts of the replica. The contributions will differ from phase to phase. The number of particles $N_{k}$ are also distributed between the phases. All contributions should be accounted for in Equation (12). The replica entropy *S* and the Gibbs energy *G* have contributions from all three phases [10]:

(13) $$S\left(T,p,N_{k},\Omega \right)=S^{c}+S^{s}+S^{e}$$

(14) $$G\left(T,p,N_{k},\Omega \right)=G^{c}+G^{s}+G^{e}$$

The superscript “*c*” stands for bulk colloidal phase, “*e*” for the bulk electrolytic phase and “*s*” for the Gibbs dividing interface. The excess Gibbs energy of the surface, $G^{s}$, is mathematically defined as the excess of *G* for a zero-width interface (the Gibbs interface) between the two bulk phases. All the differences in thermodynamic quantities between the bulk phases and the interfacial region between the two bulk phases are ascribed to excess quantities defining the zero-width dividing Gibbs surface. The bulk phases have of course no contribution from the surface. We assume that the chemical potentials inside the colloidal particle and in the solvent (water and salt ions) are the same. A recent discussion about the relation between chemical potentials inside and outside a colloidal particle (in this case a crystallite) can be found in [25].

Following Hill, we now introduce the *integral surface tension* $\hat{\gamma }$ to deal with the system smallness:

(15) $$\hat{\gamma }\Omega \equiv \gamma \Omega +\varepsilon$$

If we expand the surface area by adding one replica to the ensemble, there is a contribution to $G^{s}$ from γΩ and from the subdivision potential, ε. This leads to the definition of the integral surface tension $\hat{\gamma }$, following Equation (15). If, on the other hand, we increase the surface energy by stretching each colloidal particle surface in the ensemble, there is only one contribution, that from the differential surface tension γ. When Equation (15) is introduced into Equation (10), we obtain the *excess Gibbs surface energy*:

(16) $$\begin{array}{c} G^{s}=\mu_{k}N_{k}^{s}+\hat{\gamma }\Omega \end{array}$$

The chemical potential is a control variable and does not obtain the superscript *s*. Terms containing the standard state in $G^{s}$ and $\mu_{k}$ cancel. In the present case, there is one excess component *k*, namely the unpolarized molecules of the colloidal particles at the colloidal surface. Without loss of generality, we are going to assume that these particles are made of a certain amount of polymer RM, and hence, we write $N^{s}=N_{\mathrm{RM}}^{s}$. We will investigate the case where the polymer at the particle/electrolyte interface can dissociate according to RM = R^+^ + M^−^ in Section 3. Equation (16) gives the integral surface tension

(17) $$\hat{\gamma }=\frac{G^{s}-\mu_{\mathrm{RM}}N_{\mathrm{RM}}^{s}}{\Omega }$$

In both Equations (15) and (17), the integral surface tension is a function of the temperature and the pressure, compatible with the use of the Gibbs excess energy. The integral surface tension is an absolute quantity, like the differential surface tension.

The differential of $G^{s}$ in Equation (16) is

(18) $$dG^{s}=\mu_{\mathrm{RM}}dN_{\mathrm{RM}}^{s}+N_{\mathrm{RM}}^{s}d\mu_{\mathrm{RM}}+d\left(\hat{\gamma }\Omega \right)$$

We compare this expression for $dG^{s}$ with Equation (11), written for the surface, and obtain

(19) $$\begin{array}{c} d\left(\hat{\gamma }\Omega \right)=-S^{s}dT-N_{\mathrm{RM}}^{s}d\mu_{\mathrm{RM}}+\gamma d\Omega \end{array}$$

This equation can be called the Hill–Gibbs–Duhem equation following [17]. It was used to describe the adsorption of CO_2_ at small carbon particles with constant surface area at isothermal conditions [15,16]. It now also follows that the integral surface tension in Equation (16) can be identified with $\hat{G}$ in Equation (2). When $\hat{\gamma }=\gamma$, we recover the normal Gibbs–Duhem equation for the surface, as we should.

At constant *T* and *p*, we have from Equation (19):

(20) $$d\left(\hat{\gamma }\Omega \right)=\gamma d\Omega -N_{\mathrm{RM}}^{s}d\mu_{\mathrm{RM}}$$

The Maxwell relation follows:

(21) $${\left(\frac{\partial \gamma }{\partial \mu_{\mathrm{RM}}}\right)}_{T,p,\mu_{\mathrm{salt}},\mu_{w}}=-{\left(\frac{\partial N_{\mathrm{RM}}^{s}}{\partial \Omega }\right)}_{T,p,\mu_{\mathrm{salt}},\mu_{w}}$$

The surface tension γ will change with changing chemical potential $\mu_{\mathrm{RM}}$, depending on the excess concentration of RM.

In summary, it follows from the Hill–Gibbs–Duhem Equation (19) that

(22) $$\gamma \left(T,p,\mu_{k},\Omega \right)={\left(\frac{\partial (\hat{\gamma }\Omega )}{\partial \Omega }\right)}_{T,p,\mu_{k}}=\hat{\gamma }+\Omega {\left(\frac{\partial \hat{\gamma }}{\partial \Omega }\right)}_{T,p,\mu_{k}}$$

This is the Shuttleworth(-like) equation in its most general form, as derived using the concepts of thermodynamics for small systems. Equation (22) has the same form as the equation given by Shuttleworth in 1950 [1]. Shuttleworth gave a relation between the excess surface Gibbs energy density and the tensorial surface tension of a crystalline solid (the surface stress). Our relation applies to scalars, and we do not consider stress in crystalline structures. Importantly, we specify the variables to keep constant in the experiment. In our case, the difference between γ and $\hat{\gamma }$ is due to their dependence on the surface curvatures. In Hill’s description, both are thermodynamic properties.

Equation (22) expresses the link between the integral and differential surface tensions. It can be connected with the subdivision potential:

(23) $$\varepsilon \left(T,p,\mu_{k},\Omega \right)=\Omega \left(\hat{\gamma }-\gamma \right)=-\Omega^{2}{\left(\frac{\partial \hat{\gamma }}{\partial \Omega }\right)}_{T,p,\mu_{k}}$$

The subdivision potential is the energy needed for division of systems into smaller parts. The relation to the integral and differential surface tension is discussed below for a particular example where curvature energy is involved.

### 2.2. The Differential and Integral Surface Tension of a Curved Surface

Equations (21)–(23) were derived using the thermodynamic theory for small systems. Two types of surface tensions (the integral and the differential surface tensions) were introduced, defined by Equations (12) and (15). Once we know one of these surface tensions, we can determine the other using the aforementioned equations.

To illustrate the relation between the surface tensions, consider a colloid particle of arbitrary shape with radii of curvature $a_{1}$ and $a_{2}$; see, e.g., [26] for analysis of experiments. The curvature *C* is

(24) $$C\equiv \frac{1}{a_{1}}+\frac{1}{a_{2}}$$

meaning that *C* is constant for a sphere of radius *a*, C=2/a. The Gaussian curvature is

(25) $$C_{G}\equiv \frac{1}{a_{1}a_{2}}$$

giving $C_{G}=1/a^{2}$ for the sphere.

The spherical particle has volume $V^{c}$ and surface area $\Omega =4\pi a^{2}$. The relations between the curvatures and Ω become

(26) $$\Omega =\frac{16\pi }{C^{2}}=\frac{4\pi }{C_{G}}$$

For flat surfaces, *C* and $C_{G}$ are zero.

The energy represented by the curvature of the sphere is a reason why the integral and the differential surface tensions differ from one another. We expand the functional dependence of the integral surface tension in terms of curvatures and find [27,28,29]

(27) $$\hat{\gamma }\left(C,C_{G}\right)=\gamma_{0}+\frac{1}{2}KC\left(C-2C_{0}\right)+K_{G}C_{G}$$

Here, $\gamma_{0}$ is the surface tension for a flat surface, and *K* and $K_{G}$ are the rigidity moduli. For colloids and micro-emulsions, *K* is positive [30]. It is likely that $\hat{\gamma }$ is a function of the Gaussian curvature $C_{G}$. We do not expect γ, which is connected to surface stretching, to be such a function (see below).

When $K_{G}$ = 0, the integral surface tension $\hat{\gamma }$ has a minimum. This occurs when the curvature is equal to the system’s natural curvature, $C=C_{0}=2/a_{0}$. An increase in the curvature will reduce $\hat{\gamma }\left(C\right)-\gamma_{0}$, as long as $0<C<C_{0}$, and then increase it.

The function $\hat{\gamma }\left(C\right)-\gamma_{0}$ becomes positive for $C=2C_{0}$, the curvature for which $\hat{\gamma }\left(C\right)=\gamma_{0}$. The curvature corresponding to the minimum energy of the whole surface of the sphere can be found by minimizing $\hat{\gamma }\Omega$. Using Equations (26) and (27) for $K_{G}$ = 0, we find that this curvature is given by $C_{min}=2\gamma_{0}/\left(KC_{0}\right)$.

By differentiating $\hat{\gamma }\Omega$ with respect to Ω, we obtain the differential surface tension as a function of *C*.

(28) $$\gamma \left(C\right)=\gamma_{0}-\frac{1}{2}KC_{0}C$$

Both $\hat{\gamma }$ and γ are second order polynomials in the curvature *C*. The $\hat{\gamma }$ is linear in $C_{G}$, while γ does not depend on $K_{G}$ as one may expect. This supports the idea of using the Helfrich expansion for $\hat{\gamma }$ rather then for γ. From Equation (28), we find, for the Tolman length, [27,31]

(29) $$\delta_{T}=\frac{KC_{0}}{\gamma_{0}}=\frac{2K}{a_{0}\gamma_{0}}$$

The Tolman length has the same sign as $C_{0}$. We refer to Blokhuis et al. [32] for a discussion on the sign of $\delta_{T}$. We note in passing that γ and $\hat{\gamma }$ do not follow Hadwigers theorem; see [13,33] and discussions therein (Hadwiger’s theorem would give K=0).

**Figure 3:**
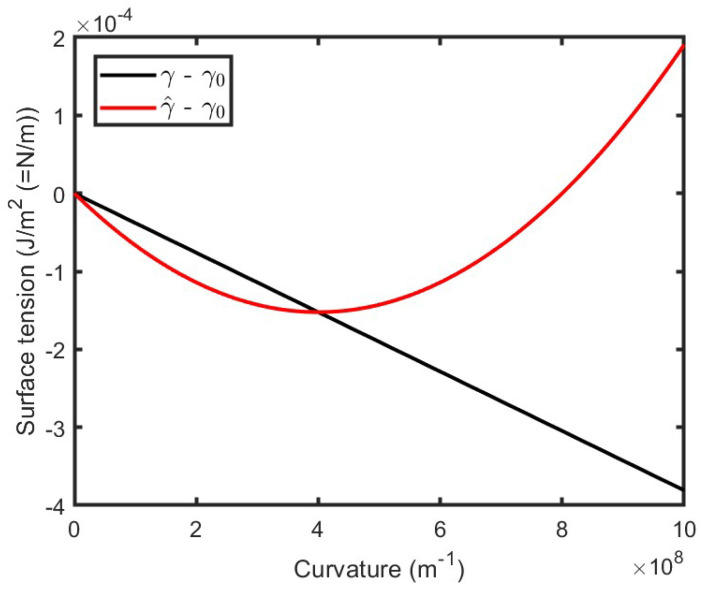
The differential (black curve) and integral (red curve) surface tension of a spherical particle, as a function of particle curvature. A natural curvature $C_{0}=4\times 10^{8}$ m^−1^ was chosen, corresponding to a particle of radius 5 nm. The rigidity modulus was chosen to be $K=0.46\;k_{B}T$, in accordance with the values found in [30].

Both γ and $\hat{\gamma }$ are illustrated in Figure 3. The figures were plotted for $K_{G}=0$ using the value of *K* found in [30] for a colloidal particle having a natural curvature of $C=4\times 10^{8}$ m^−1^, corresponding to a particle with radius a=5 nm. The rigidity modulus was chosen to be $K=0.46\;k_{B}T$. The black curve represents $\gamma -\gamma_{0}$ and is a negative function (see Equation (27)). The red curve $\hat{\gamma }-\gamma_{0}$ is is a parabolic function of *C*. The difference between $\gamma -\gamma_{0}$ and $\hat{\gamma }-\gamma_{0}$ can be understood by studying the subdivision potential (see Equation (15)), which is done in the following subsection.

### 2.3. The Subdivision Potential and Its Scaling Law

**Figure 4:**
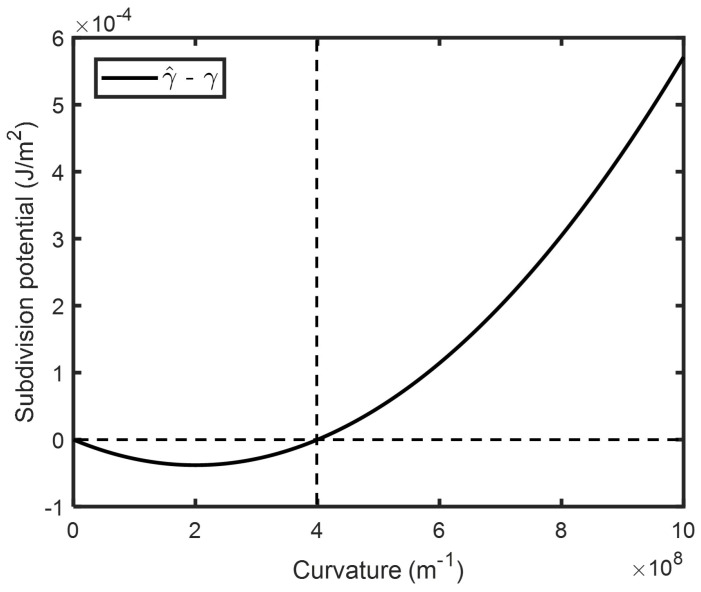
The subdivision potential per unit of surface area of a spherical particle as a function of particle curvature. The natural curvature $C_{0}=4\times 10^{8}$ m^−1^ was chosen to correspond to a particle of 5nm radius, and the rigidity modulus was chosen to be K=0.46 $k_{B}T$, in accordance with the values found in [30]. We see that $\hat{\gamma }<\gamma$ when *C* is smaller than the natural curvature. In this range, the system will tend to change its curvature to the natural one.

The subdivision potential ε is a trademark of the thermodynamics of small systems [17]. It describes, using ensemble theory, the effect of system smallness on the system energy and characterizes the system’s deviation from Euler homogeneity. It accounts for the ability of increasing the total surface area made of the sum of all the colloidal particle surface areas in the ensemble of replicas. This can be done in two ways (cf. Figure 2): (1) by stretching the area of each colloidal particle in the ensemble, keeping the pressure *p* as well as the number of replica’s constant, or (2) by increasing the number of replicas in the ensemble, keeping the pressure and the surface area of each colloidal particle constant. An illustration of the subdivision potential per unit of surface area and its link to the differential and integral surface tension is shown in Figure 4. By rearranging Equation (23), we find what can be called a system scaling law. We obtain

(30) $${\left(\frac{\partial \hat{\gamma }}{\partial \Omega }\right)}_{T,p,\mu_{k}}=-\frac{\varepsilon }{\Omega^{2}}$$

Figure 4 shows that ε is negative (unstable droplet) for $C<4\times 10^{-8}$. It becomes positive (stable droplet) for larger values.

For a spherical colloidal particle, this gives

(31) $${\left(\frac{\partial \hat{\gamma }}{\partial C}\right)}_{T,p,\mu_{k}}=\frac{C}{8\pi }\varepsilon$$

From Equations (27) and (28), we obtain the subdivision potential per unit of surface area. This can be called the system’s scaling law:

(32) $$\frac{\varepsilon }{\Omega }=\hat{\gamma }-\gamma =\frac{1}{2}KC\left(C-C_{0}\right)+K_{G}C_{G}$$

The curve starts at ε=0 and $C=C_{G}=0$. For $K_{G}=0$ and $C<C_{0}$, the subdivision potential is negative. According to Equation (31), $\hat{\gamma }$ is then a decreasing function of curvature. This means that the colloidal particle becomes increasingly more stable, as it reduces in size until it reaches the natural curvature.

For small curvatures (large radii), the difference in the two surface tensions is linear and given by $-KCC_{0}/2$. Such a scaling law of $\hat{\gamma }-\gamma$ with the inverse of the particle’s radius could be proven experimentally. Scaling with the inverse system size, 1/*L*, was used to compute thermodynamic factors, central to Kirkwood–Buff integrals in solution theory [34,35]. The exact scaling law depends on the ensemble in question. For examples of scaling laws in other ensembles, see [17].

### 2.4. Alternative Variable Sets

**Figure 5:**
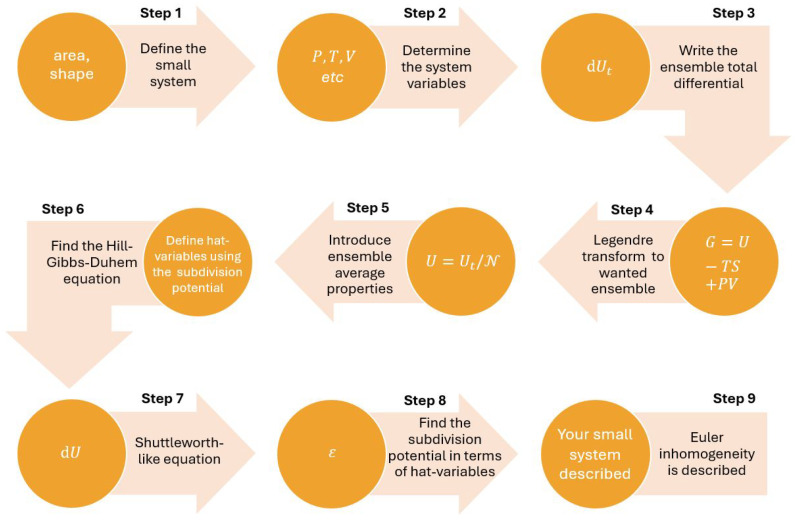
Procedure for construction of the subdivision potential in nanothermodynamics for a system which is not Euler homogeneous of the first order in size.

The thermodynamic theory for small systems applies naturally to other sets of variables than those used so far. The subdivision potential corresponding to the set can be defined following a systematic procedure. The steps to proceed are given in Figure 5.

In the system pictured in Figure 1 (the replica), we took the pressure and the surface area, Ω, as variables. Now, we choose another example where the volume *V* rather than the pressure *p* is a variable. The procedure starts by defining the small system in **Step 1**. In **Step 2**, we take *V* rather than *p* as a system variable. The variables are then $T,V,N_{k}$ and Ω.

**Step 3** gives the Hill–Gibbs equation for the ensemble of replicas $dU_{t}$; see Equation (5). The subdivision potential is introduced through this total differential. On the ensemble level, we next do the Legendre transform to deal with the properties of interest (**Step 4**). In Section 2, we chose to go from $U_{t}$ to $G_{t}$. In the new example, we transform to the total Helmholtz energy, $F_{t}$. For the ensemble of small systems, we have

(33) $$F_{t}\equiv U_{t}-TS_{t}$$

The Hill–Gibbs equation corresponding to Equation (9) becomes

(34) $$dF_{t}=-S_{t}dT-pdV_{t}+\mu_{k}dN_{k,t}+\gamma d\Omega_{t}+\varepsilon dN$$

This equation with the subdivision potential is integrated here to give the Euler equation:

(35) $$\begin{array}{cc} F_{t} & =-pV_{t}+\mu_{k}N_{k,t}+\gamma \Omega_{t}+\varepsilon N\\ & =-pV_{t}+\mu_{k}N_{k,t}+\hat{\gamma }\Omega_{t} \end{array}$$

The expression corresponds to Equation (8). The ensemble average properties can now be introduced, **Step 5**, similarly to what was done in Equation (10).

(36) $$\begin{array}{cc} F & =-pV+\mu_{k}N_{k}+\gamma \Omega +\varepsilon \\ & =-pV+\mu_{k}N_{k}+\hat{\gamma }\Omega \end{array}$$

By introducing the averages, we obtain the Gibbs equation:

(37) $$dF=-SdT-pdV+\mu_{k}dN_{k}+\gamma d\Omega$$

The definition of the differential surface tension follows:

(38) $$\gamma \left(T,V,N_{k},\Omega \right)={\left(\frac{\partial F}{\partial \Omega }\right)}_{T,V,N_{k}}$$

The definition underscores the importance of the control variables.

The Hill–Gibbs–Duhem equation (**Step 6**) is

(39) $$d\left(\hat{\gamma }\Omega \right)=-SdT+Vdp-N_{k}d\mu_{k}+\gamma d\Omega$$

The Shuttleworth-like equation follows, **Step 7**, and is given by

(40) $$\gamma ={\left(\frac{\partial (\hat{\gamma }\Omega )}{\partial \Omega }\right)}_{T,p,\mu_{k}}=\hat{\gamma }+\Omega {\left(\frac{\partial \hat{\gamma }}{\partial \Omega }\right)}_{T,p,\mu_{k}}$$

The equation is the same as Equation (22), with the same experimental conditions. The subdivision potential can consequently be given (**Step 8**):

(41) $$\varepsilon =-\Omega^{2}{\left(\frac{\partial \hat{\gamma }}{\partial \Omega }\right)}_{T,p,\mu_{k}}$$

This is identical to Equation (23). **Step 9** completes the description of the non-homogeneous system by nanothermodynamics.

Yet another Shuttleworth equation can be obtained for different variables. For example, for an ensemble with constant variables $T,p,\mu_{k}$ and a variable line length, we obtain

(42) $$\lambda ={\left(\frac{\partial (\hat{\lambda }L)}{\partial L}\right)}_{T,p,\mu_{k}}=\hat{\lambda }+L{\left(\frac{\partial \hat{\lambda }}{\partial L}\right)}_{T,p,\mu_{k}}$$

Here, *L* is the line length, and λ is the line tension. Bedeaux et al. gave an overview of the subdivision potential for six common ensembles [13]. The procedure summarized here does not apply to colloids only but is also available for numerous systems that qualify as being “small”, in the sense that they do not possess Euler homogeneity.

## 3. Surface Polarization

The thermodynamic state of the surface of a large class of systems can be altered by application of an electric field. This electric field can be externally applied or caused by an electric charge distribution inside the system. It is known that such an electric field can change the surface tension of a system [11], and this property, called electrowetting, has been used in microfluidic applications [36]. The presence of an externally applied electric field leads to charge separation inside a polarizable region.

We shall see that this can be modeled and understood by dividing such a region of space into two layers, s1 and s2. The layer s1 has a net charge $Q^{s1}=Q$, while the layer s2 has the net charge $Q^{s2}=-Q$. The layer s=s1+s2 is, however, electroneutral. The separation between net charges, the polarization, represents surface energy. It is possible to define a Gibbs zero-width dividing interface for *s*. While the charges depend on the choice of this dividing surface, the polarization does not, due to electroneutrality. An excess surface internal energy $P^{s}D^{d}/2\varepsilon_{0}\varepsilon_{1}$ can therefore be defined and associated with the Gibbs surface, $P^{s}$, independent of the surface position. Here, $D^{d}$ is the displacement field normal to the surface and is constant through the surface (see [37], p. 76), $P^{s}$ is the excess polarization normal to the surface (the integral over the surface of the normal component of the polarization), and the dielectric permittivity of the interfacial region s2 is chosen to be $\varepsilon_{0}\varepsilon_{1}$, where $\varepsilon_{0}$ is the dielectric permittivity of the vacuum and $\varepsilon_{1}$ the relative permittivity of the solvent. The origin of the factor 2 will be explained at the end of the section, where we calculate the electric energy of the diffuse layer. The energy due to $P^{s}$ needs be added to the other parts of the excess Gibbs surface energy, $dG^{s}$, in Equations (17) and (18) to give the equation:

(43) $$d{\tilde{G}}^{s}=-S^{s}dT-\Omega d\gamma +{\tilde{\mu }}_{j}^{s}dN_{j}^{s}+\frac{P^{s}}{2\varepsilon_{0}\varepsilon_{1}}dD^{d}$$

which is the Gibbs equation. Here ${\tilde{G}}^{s}=G^{s}+P^{s}D^{d}/2\varepsilon_{0}\varepsilon_{1}$, and the electric field in the interfacial region s2 is $D^{d}/\left(\varepsilon_{0}\varepsilon_{1}\right)$. While Equation (43) seems to belong to classical thermodynamics, we know from the present analysis that the system does not possess Euler homogeneity in the surface area variable. The variables of Equation (43) may therefore differ from their thermodynamic limit values. We shall see below how the charge density can replace $P^{s}$ in Equation (43).

We consider an ensemble of replicas made of charged colloidal spheres at constant temperature, pressure and chemical potentials of the electrolyte. The electrochemical potential of the ion *j*, ${\tilde{\mu }}_{j}$, is defined by ${\tilde{\mu }}_{j}=\mu_{j}+z_{j}F\psi$, where $\mu_{j}$ is the chemical potential of the ion, $z_{j}$ is the charge number, *F* is Faraday’s constant, and ψ is the Maxwell potential.

The excess properties of the Gibbs dividing interface are not Euler homogeneous in the surface area. The system is therefore small in the sense that we have defined it upfront (relatively large surface energy on an energy scale). From Equations (10) and (14), we obtain

(44) $${\tilde{G}}^{s}={\tilde{\mu }}_{j}N_{j}^{s}+\varepsilon$$

The Euler inhomogeneity of ${\tilde{G}}_{j}^{s}$ means that $\hat{\gamma }\neq \gamma$. By differentiating this equation and subtracting the Hill–Gibbs equation Equation (43), we obtain a Hill–Gibbs–Duhem equation:

(45) $$d\left(\hat{\gamma }\Omega \right)=-S^{s}dT+\gamma d\Omega -N_{j}^{s}d{\tilde{\mu }}_{j}+\frac{P^{s}}{2\varepsilon_{0}\varepsilon_{1}}dD^{d}$$

Equations (43) and (45) will lead to the Lippman equation.

### 3.1. A Polarizable Colloidal Particle

**Figure 6:**
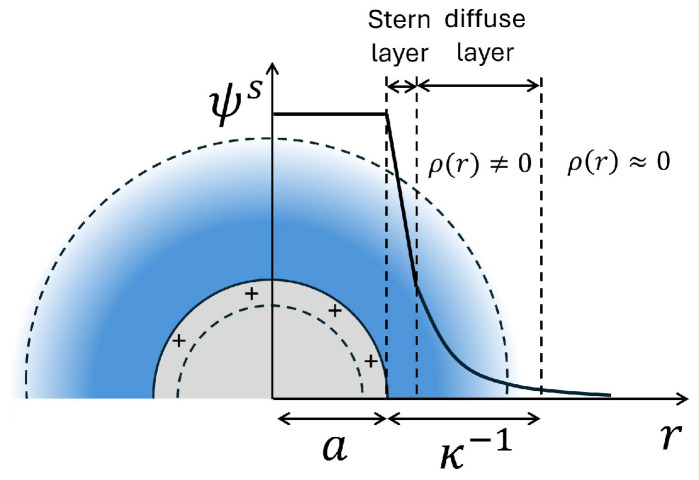
Schematic view of a colloidal particle with double layer. The figure shows the fixed positive charges grafted on the surface of the particle. The colloidal phase is grey, and the volume that contains the ionic charges of the diffuse layer is in shades of blue. The electrostatic potential inside the particle is constant in the absence of an external electric field and equal to the surface electric potential, $\psi^{0}$. The thickness of the Stern layer is greatly exaggerated.

We model the case of a charged colloidal particle in an electrolyte solution and derive a Lippman equation using Equations (43) and (45). The charged colloidal particle in the electrolyte solution has an ionic *diffuse layer* that extends from its surface into the solvent. The situation is illustrated in Figure 6. We consider a spherical colloidal particle with a fixed surface charge Q>0 in layer s1 (gray area at the surface of the particle) and a charge Q<0 in layer s2 (area in shades of blue). Layer s2 is called the diffuse layer. The combined layer s=s1+s2 is called the *double layer*. The colloidal particle has *N* surface sites that are dissociated according to RM = R^+^ + M^−^. The surface charge density is taken to be homogeneous. The counter-ions $M^{-}$ will be part of the double layer, but we emphasize that the diffuse layer also contains ions from the electrolyte, with, in the present case a majority of anions (although cations are also present in the diffuse layer). The concentration of electrolytes is directly related to the thickness of the double layer (called the Debye length); see Equation (56). The diffuse layer is not electroneutral, and the integrated charge over the diffuse layer is −Q. Beyond the double layer, electroneutrality applies. In the figure, an additional layer (Stern layer) is illustrated. In this region of space, ions can be partially hydrated and experience a chemical or physical sorption with the surface of the particle. This layer will not be further discussed in the present article, and the colloidal particle will be assumed to be “ideal” and devoid of the Stern layer. Consequently, we will assume that the Poisson–Boltzmann relation applied directly from the surface of the particles. We refer to Verwey and Overbeek [11] and Debye [38,39] for a thorough description of charged colloidal systems and Stern layers in particular. The figure shows that the electrostatic potential at equilibrium decreases from the surface value $\psi^{s}$ to a solution value of zero. Due to the spherical symmetry of the system, the electric potential ψ and the charge density, ρ, depend only on the distance *r* from the center of the colloidal particle.

#### 3.1.1. Layer *s*1

The innermost layer contains the fixed charged surface groups. We will now consider a zero-width dividing Gibbs interface inside s1, which we link with an excess charge density. with the thickness of s1, the Gibbs interface can be taken to coincide with the particle/electrolyte interface. The excess charge density $\rho^{s}$ (C/m^2^) is defined by

(46) $$Q^{s}=FN_{R^{+}}^{s}=\rho^{s}\Omega$$

The electrochemical potential of R^+^ is everywhere constant, giving

(47) $$\mu_{R^{+}}^{s1}=-z_{R^{+}}F\psi^{s}$$

where we used the fact that the electrochemical potential $\tilde{\mu }$ reduces to the chemical potential μ beyond the double layer, ${\tilde{\mu }}_{R^{+}}\left(\infty \right)=\mu_{R^{+}}\left(\infty \right)$, taking the standard chemical potential $\mu_{R^{+}}^{0}=0$. This equation has as a consequence that there is no contribution to the excess compressional energy ($\hat{\gamma }\Omega$) from the surface ions $R^{+}$ in *s*1 and, therefore, also not to the surface tension [11]. Any change in electric energy will be compensated by a corresponding change in the chemical potential. The excess electric energy contribution to the surface tension of the system is then given solely by the diffuse layer *s*2. The Gibbs energy for *s*1 is equal to $G^{s1}=U^{s1}-TS^{s1}$ and ${\tilde{G}}^{s1}=G^{s1}+Q\psi^{s}$. We combine and obtain

(48) $$d{\tilde{G}}^{s1}=-S^{s1}dT+Qd\psi^{s}$$

The layer variables $S^{s1}$, *Q* and $G^{s1}$ are the excess variables of the surface.

#### 3.1.2. Layer *s*2

This framework is identical to the common thermodynamic framework of the electrical double layer, with $s=s_{1}+s_{2}$ (fixed charge layer + diffuse layer). Polarization means that there is charge separation. The surface charge in s1 on the colloidal particle, *Q*, is equal to minus the excess charge of the diffuse layer around the colloidal particle in s2 [11]:

(49) $$Q=-\int_{a}^{\infty }4\pi r^{2}\rho \left(r\right)dr$$

The well-known model of Debye and Hückel will now be used to relate $P^{s}$ and *Q*, as computed from Equation (49). The infinity in the integral indicates that we have chosen s2 such that the whole diffuse layer is contained, but the ionic densities reach their bulk values before the border of the replica. The electroneutrality condition holds in the bulk phase far away from the charged colloid, giving

(50) $$\sum_{i}\nu_{i}z_{i}=0$$

The ions (i=+,−) have a valence $z_{i}$, and their stoichiometric coefficient is $\nu_{i}$. The charge density, ρ, is a function of the distance *r* from the colloidal particle is expressed by

(51) $$\rho \left(r\right)=\sum_{i}z_{i}Fc_{i}\left(r\right)$$

where $c_{i}$ is the molar density of component *i*. The ionic concentrations follow the Boltzmann distribution:

(52) $$c_{i}=\nu_{i}C^{s}\exp\left(-z_{i}e\psi \left(r\right)/k_{B}T\right)$$

Here, $C^{s}$ is the salt concentration in mM (10^−3^ mol/L), *e* is the elementary charge, $k_{B}$ is the Boltzmann constant, and *T* is the temperature. Outside the colloidal particle, the potential satisfies the Poisson equation

(53) $$\Delta \psi \left(r\right)=\frac{\partial^{2}\psi \left(r\right)}{\partial r^{2}}+\frac{2}{r}\frac{\partial \psi (r)}{\partial r}=-\frac{\rho (r)}{\varepsilon_{0}\varepsilon_{1}}$$

where $\varepsilon_{0}$ is the permittivity of the vacuum, and $\varepsilon_{1}$ is the relative permittivity of the solvent (water).

For small electric potentials, combining Equations (51)–(53), the charge density is given by the Debye–Hückel relation [38,39]

(54) $$\rho \left(r\right)=-\varepsilon_{0}\varepsilon_{1}\kappa^{2}\psi \left(r\right)$$

where the resulting potential distribution is

(55) $$\psi \left(r\right)=\frac{a}{r}\psi^{s}\exp\left(\kappa \left(a-r\right)\right)$$

and the characteristic length scale of the diffuse layer s2 is given by the Debye length [38,39]:

(56) $$\kappa^{-1}=\sqrt{\frac{\varepsilon_{0}\varepsilon_{1}RT}{F^{2}C^{s}\sum z_{i}^{2}\nu_{i}}}$$

where *R* is the gas constant. The salt concentration is sufficiently large so that the contribution of the dissociated ions to the Debye length can be neglected.

The electric potential at the surface of the colloid is taken as a boundary condition. It is given by

(57) $$\psi \left(a\right)=\psi^{s}$$

The potential inside the colloidal particle is equal to $\psi^{s}$ by continuity. An alternative boundary condition would be to impose a surface charge density (Q/Ω) at the surface. For a single colloidal particle in an electrolyte, the choice makes no difference. Increasing the surface charge density (for a given ionic strength) will automatically lead to a change in electric potential and vice-versa. Only when colloidal particles approach one other do we need to distinguish between conditions of constant charge and constant potential.

When these relations are introduced into the expression for *Q*, we obtain

(58) $$\begin{array}{ccc} Q & = & 4\pi \varepsilon_{0}\varepsilon_{1}\kappa^{2}\int_{a}^{\infty }r^{2}\psi \left(r\right)dr=4\pi \varepsilon_{0}\varepsilon_{1}\kappa^{2}a\psi^{s}\int_{a}^{\infty }r\exp\left(\kappa (a-r)\right)dr\\ & = & 4\pi a^{2}\varepsilon_{0}\varepsilon_{1}\kappa \psi^{s}\left(1+\frac{1}{\kappa a}\right)=\Omega \rho^{s} \end{array}$$

where $\rho^{s}$ is the density of the surface charges of the colloid surface and

(59) $$\rho^{s2}=-\rho^{s1}\equiv -\rho^{s}$$

The electric energy of the diffuse layer s2 is

(60) $$\begin{array}{cc} 4\pi \int_{a}^{\infty }r^{2}\rho \left(r\right)\psi \left(r\right)dr= & -4\pi a^{2}\varepsilon_{0}\varepsilon_{1}\kappa^{2}{\left(\psi^{s}\right)}^{2}\int_{a}^{\infty }\exp\left(2\kappa \left(a-r\right)\right)dr\\ = & -\frac{1}{2}\Omega \varepsilon_{0}\varepsilon_{1}\kappa {\left(\psi^{s}\right)}^{2}\approx -\frac{1}{2}Q\psi^{s} \end{array}$$

In the derivation, we used κa>>1. Furthermore, we have

(61) $$\begin{array}{cc} 4\pi \int_{a}^{\infty }r^{2}\rho \left(r\right)d\psi \left(r\right)dr= & -2\pi \varepsilon_{0}\varepsilon_{1}\kappa^{2}\int_{a}^{\infty }r^{2}d\left(\psi^{2}\left(r\right)\right)dr\\ = & -2\pi \varepsilon_{0}\varepsilon_{1}\kappa^{2}d\int_{a}^{\infty }r^{2}\left(\psi^{2}\left(r\right)\right)dr\\ = & -2\pi a^{2}\varepsilon_{0}\varepsilon_{1}\kappa \psi^{s}d\psi^{s}\approx -\frac{1}{2}Qd\psi^{s} \end{array}$$

where we again used κa>>1.

We combine the expressions to give the electric energy of the whole double layer, s=s1+s2, $\frac{1}{2}Q\psi^{s}$. We observe that the energies of s1 and s2 have opposite signs and that the presence of diffuse layer lowers the total energy and thereby stabilizes s1. The differential of the electric energy of the double layer becomes $-\frac{1}{2}Qd\psi^{s}$. The Gibbs equation for s2 is accordingly

(62) $$d{\tilde{G}}^{s2}=d\left(G^{s2}-\frac{1}{2}Q\psi^{s}\right)=-S^{s2}dT-\Omega d\gamma +{\tilde{\mu }}_{k}dN_{k}^{s2}-\frac{1}{2}Qd\psi^{s}$$

The summation is over all ions in s2. The permanently charged sites of the polymer R^+^ are contained in s1. Furthermore, $G^{s2}\equiv U^{s2}-TS^{s2}-\gamma \Omega$.

The expression for the whole layer s=s1+s2 becomes

(63) $$d{\tilde{G}}^{s}=d\left(G^{s}+\frac{1}{2}Q\psi^{s}\right)=-S^{s}dT-\Omega d\gamma +\mu_{k}dN_{k}^{s}+\frac{1}{2}Qd\psi^{s}$$

The derivation explains the factor 2 in the denominator, as promised upfront. The total surface is electroneutral, and the sum is therefore over the excess amounts of the neutral species. The electrochemical potentials reduce to the chemical potentials. The equation above may seem to belong to the classical thermodynamic theory. It should be remembered once more, however, that the variables in Gibbs’ equation here are not Euler homogeneous! They will, therefore, not obey all classical thermodynamic formulas.

### 3.2. The Lippman Equation

In the previous subsection, we found the excess properties of the model layer s=s1+s2 as a sum of the corresponding excess properties in the s1 and s2 layers. The total surface was electroneutral. Subsystems s1 and s2, however, both have a net charge. In our example, s1 has a positive excess charge, *Q*, while the diffuse layer, s2, has a negative excess charge, −Q.

It follows from Equation (62) under these conditions that

(64) $${\left(\frac{\partial \gamma }{\partial \psi^{s}}\right)}_{T,p,{\tilde{\mu }}_{k}}=-\frac{1}{2}{\left(\frac{\partial Q}{\partial \Omega }\right)}_{T,p,{\tilde{\mu }}_{k}}=-\frac{1}{2}\rho^{s}$$

Equation (64) has the form of a Lippmann equation [2,11]. By following the procedure defined in Figure 5, we imposed the temperature *T*, the pressure *p* and the electrochemical potential ${\tilde{\mu }}_{k}$ to be kept constant when varying the surface area Ω. By integrating Equation (64), using Equation (38) and taking κa>>1, which gives $\rho^{s}=\varepsilon_{0}\varepsilon_{1}\kappa \psi^{s}$, we obtain

(65) $$\gamma -\gamma_{0}=-\frac{\varepsilon_{0}\varepsilon_{1}\kappa }{4}{\left(\psi^{s}\right)}^{2}$$

where $\gamma_{0}$ applies to a surface without excess electric charge. The same formula was obtained by Vis and Blokhuis [40] for a demixed polymer solution. We see that $\gamma <\gamma_{0}$. It is well known [11] that the charging of a surface leads to a smaller surface tension. The value $\gamma_{0}$ can be ascribed, e.g., to van der Waals-type forces.

The polarization can be calculated from

(66) $$P^{s}=4\pi \int_{a}^{\infty }\left(r-a\right)\rho \left(r\right)r^{2}dr=-\Omega \frac{\rho^{s}}{\kappa }\frac{\kappa a+2}{\kappa a+1}\approx -\frac{Q}{\kappa }$$

The polarization is negative with positive charges in s1 and negative charges in s2 (the diffuse layer). The displacement field (in the normal direction) is given by

(67) $$\frac{D(r)}{\varepsilon_{0}\varepsilon_{1}}=-\frac{\partial \psi (r)}{\partial r}=\kappa \left(1-\frac{1}{r\kappa }\right)\psi \left(r\right)\approx \kappa \psi \left(r\right)$$

Furthermore, $D^{d}\equiv D\left(a\right)$. It follows for the diffuse layer that

(68) $$-\frac{1}{2}Q\psi^{s}=-\frac{P^{s}}{2\varepsilon_{0}\varepsilon_{1}}D^{d}$$

Because the energy of the adsorbed layer plus the diffuse layer is equal to minus the energy of the diffuse layer, the relations explain the added electric energy in Equation (43). Mugele [41], page R755, mentions two contributions from the surface electric energy: one from a parallel plate capacitor and another from stray capacitance at the droplet edge. The term $Q\psi^{s}$ contains such capacitance contributions.

Lippmann in his original experimental work related the change in the surface tension due to an external electric field by a change in the surface charge density. His system was a surface between a mercury drop and an electrolyte [41]. The system in the present study is not subject to an external electric field, but the Lippmann equation applies also to a polarized surface of a colloidal particle. Lagrange transformations were used to deal with constant voltage conditions rather than constant charge. Such transformations can be done in nanothermodynamics on the ensemble level (i.e., variables with subscript *t*).

## 4. Discussion

### 4.1. Choice of Variables

The variables in Hill’s thermodynamics for small systems depend on the environmental variables used to control the system (the *control variables*). In classical thermodynamics, this is not the case. A system property does not depend on the ensemble used in the measurement. The equation of state, e.g., does not vary with the chosen ensemble for a system that is Euler homogeneous of the first order in the system size. In our example, the description is classical when $C=0,C_{G}=0$.

Hill’s method was made to deal with a system’s lack of Euler homogeneity. The method may appear cumbersome, but the extra effort is rewarded by the appearance of a series of new relations (i.e., scaling laws). In this case, like in other cases [19], these laws describe the impact of system curvatures on the variables.

The surface tension γ, termed the differential surface tension in this article, is the surface tension traditionally referred to in classical thermodynamics. We have seen here that Hill’s method can relate changes in the differential surface tension to other surface properties. The outcome of the derivation was interesting. The Shuttleworth as well as the Lippman equations followed from the same Hill–Gibbs–Duhem equation. The Hill–Gibbs equation differs from the Gibbs equation, because it deals with variables that are not Euler homogeneous, while homogeneity is a trademark of classical thermodynamics. The surface tension and the subdivision potential depend on the surface area. The methods of Hill and Gibbs are equivalent for $C=0,C_{G}=0$, which is the trivial case of the thermodynamic limit. The methods have also been found to be equivalent for systems with constant curvature [18]. The system variables were then chosen in a manner different from here: the curvature was not taken to be independent of the volume. The results reported in [18] have, therefore, no direct bearing on the findings reported here.

Hill’s method as proposed here can in principle be applied to particles of all sorts, such as colloids, micelles, droplets and bubbles for which the volume, the surface area, and the adsorptions are independently controlled. The methodology is particularly interesting for complementing the study of nucleation, a typical example of a non-Euler homogeneous system [42,43].

### 4.2. Trademarks of Thermodynamics for Small Systems

For a system in the grand canonical ensemble, we have seen that the integral surface tension $\hat{\gamma }$ is the *surface excess compressional energy density*. The integral surface tension depends on the colloid surface curvatures (*C* = 2/*a* and $C_{G}$ = 1/$a^{2}$ for a sphere) and differs from the (classical) differential surface tension γ ($\gamma \neq \hat{\gamma }$). The differential surface tension γ is related to the making of more area by reversible stretching of the area. The integral surface tension $\hat{\gamma }$, on the other hand, is the energy needed to make more surface area, not by stretching the surface, but by adding a finite area while redistributing the replica contents. The measuring conditions in each case are defined by the operations and the control variables (subscripts). When one stretches the surface, excess densities of components that are not mobile, will decrease. This makes $\hat{\gamma }$ different from γ, with the subdivision potential unequal to zero.

The nanothermodynamic theory of Hill [14], as extended by Bedeaux et al. [17], provides a method to address system energies due to the system size and curvature. Size- and curvature-related energies may be significant for systems on the micrometer scale. The method is, therefore, not limited to the nanoscale (despite the name of *nanothermodynamics* used for the thermodynamics of small systems) but can be applied in general to systems that are no longer Euler homogeneous of the first order in the size. Hill introduced a measure of system smallness through his introduction of the subdivision potential. This new property, which can be computed from the differential and integral surface tension, can be used to characterize smallness.

We have shown how the Shuttleworth and Lippman equations can be derived from the same nanothermodynamic basis, the Hill–Gibbs–Duhem equation, and that new thermodynamic potentials (ε) can be used to understand system smallness.

### 4.3. The Shuttleworth and Lippman Equations in the Literature

Equation (2) was derived by Shuttleworth in 1950 for a crystalline surface [1]. The surface tension was given in terms of the excess Helmholtz or excess Gibbs energy density, giving $G^{\prime }$, by the relation

(69) $$\gamma =G^{\prime }+\Omega \frac{dG^{\prime }}{d\Omega }$$

By comparing this equation to our Equation (22), we saw that $G^{\prime }$ may be understood as our $\hat{\gamma }$. Their meanings differ, however.

Shuttleworth made a distinction between the excess Gibbs free energy density and the surface tension. The excess surface Gibbs energy density was for him the work needed to form a unit surface area by a process of division, while the surface tension was connected to the work done due to tangential stress (force per unit length) in the layer of the crystal surface (a tensorial phenomenon). System variables were not discussed in more detail. The stress was also discussed by Kramer [44]. Equation (69) might well apply to the surface of solids, but stress has no direct connection to our analysis, in spite of the $G^{\prime }$ appearing in the same manner as $\hat{\gamma }$.

Hui and Jagota gave an overview of the debate in the literature [3]. They suggested that the difference between the surface tension and the surface energy was due to a difference in the chemical potential of the single component between the surface and bulk. They proposed a rewriting of the Shuttleworth equation to include the number of surface particles as variables. Makkonen [7] disputed this; see also Faraji et al. [6]. So far, authors [3,4,6,7,10] have failed to recognize the ensemble dependence of small system properties, and no general method to deal with Euler inhomogeneity has been suggested. This may explain some of the confusion in the field.

Discrepancies are often observed when computing the surface or interfacial tension of nanodroplets using molecular simulations. They appear when either the thermodynamical route (test-area method) and the mechanical route (virial approach) are used [45]. The underlying reason may be related to the definition of the different surface tensions (γ and $\hat{\gamma }$).

The present work proposes to use Hill’s general procedure, leading to a link between the integral and the differential surface tension. Both are scalar properties. There is no other method for a systematic derivation of this result, as far as we know. We have also seen that these properties are particular to the ensemble of interest. We have therefore identified Equation (2) by Equation (22). The form of Equation (22) follows from the expression for the Gibbs energy [17] of a replica.

To facilitate the use of nanothermodynamics, we have provided Figure 5. The figure is listing the single steps in the general procedure used to find the hat variables and the subdivision potentials. Some examples can be found in [17,19]. This is the first time that the nanothermodynamic method has been applied to describe the effect of area variations in a thermodynamic property.

## 5. Perspectives

We have described the impact of the surface area on the surface tension in a colloidal suspension. Using the nanothermodynamic theory as developed by Hill [14] and extended by Bedeaux et al. [17], we have defined and demonstrated the importance of the differential and integral surface tensions and computed their difference, which is the subdivision potential. As an example, we have demonstrated the curvature dependence of the subdivision potential ε for a small sphere in solution. The expressions are linking small system properties $\hat{\gamma }$ and ε to the curvature C=2/a in a way that can be tested by experiments. Similar functional relationships exist but remain, as far as we know, unexplored. The framework presented in this article is not limited to energetic contributions but also extends to, for example, entropy, which becomes non-additive within the Hill formalism [17].

We have shown that the internal structure of nanothermodynamics gives a link between the variables of the Shuttleworth- and the Lippman-like equations, a link arising from the lack of Euler homogeneity in the system. The range of validity of the equations, as they are understood in the thermodynamic limit, has therefore been considerably broadened. The links can be used not only to elucidate statements made in the literature but also to describe new systems. In other words, we could face a situation like Gibbs did, after the introduction of the chemical potential: we may open up possibilities to use ε for a whole new class of systems.

## Data Availability

The original contributions presented in this study are included in the article. Further inquiries can be directed to the corresponding author.
